# Carbon Monoxide Sensing Technologies for Next-Generation Cyber-Physical Systems

**DOI:** 10.3390/s18103443

**Published:** 2018-10-13

**Authors:** Turja Nandy, Ronald A. Coutu, Cristinel Ababei

**Affiliations:** Department of Electrical and Computer Engineering, Marquette University, Milwaukee, WI 53233, USA; turja.nandy@marquette.edu (T.N.); cristinel.ababei@marquette.edu (C.A.)

**Keywords:** carbon monoxide (CO), cyber-physical system (CPS), metal oxide semiconductor (MOS), microelectromechanical systems (MEMS), photoacoustic spectroscopy (PA), non-dispersive infrared (NDIR)

## Abstract

Carbon monoxide (CO) is a toxic gas, and environmental pollutant. Its detection and control in residential and industrial environments are necessary in order to avoid potentially severe health problems in humans. In this review paper, we discuss the importance of furthering research in CO sensing technologies for finding the proper material with low-range detection ability in very optimum condition. We build our discussion through the perspective of a cyber-physical system (CPS) modeling framework, because it provides a comprehensive framework to model and develop automated solutions for detection and control of poisonous chemical compounds, such as the CO. The most effective CO sensors, then, can be used in CPS network to provide a pathway for real-time monitoring and control in both industrial and household environment. In this paper, first, we discuss the necessity of CO detection, the proposal of a basic CPS framework for modeling and system development, how the CPS-CO model can be beneficiary to the environment, and a general classification of the various CO detection mechanisms. Next, a broad overview emphasizes the sensitivity, selectivity, response and recovery time, low concentration detection ability, effects of external parameters and other specifications that characterize the performance of the sensing methods proposed so far. We will discuss recent studies reported on the use of metal oxide semiconductor (MOS) sensing technologies for the detection of CO. MOS based micro-sensors play an important role in the measurement and monitoring of various trace amounts of CO gas. These sensors are used to sense CO through changes in their electrical properties. In addition to MOS based sensors, optical sensing methods have recently become popular, due to their increased performance. Hence, a brief overview of newly proposed optical based CO detection methods is provided as well.

## 1. Introduction

Carbon monoxide (CO) is a colorless, tasteless, and toxic gas to humans. It is usually the result of imperfect combustion, which creates the CO that is a great threat to human health. CO is a serious threat because it can lead to intoxication, which is one of the main causes of uncertain morbidity and mortality (mostly combustion related inhalation injury) [1,2,3,4,5]. Because of that, it is necessary to determine the best material and technology for sensing this toxic gas. Therefore, researchers investigated many materials using several methods and techniques to detect this gas and to commercialize them in the form of CO sensors. Because carbon monoxide is created in residential and household environments, it is very important to develop mini or micro sensing devices that are cost effective and efficient in these environments. Metal oxide semiconductor (MOS) based sensing of CO has received a lot of attention because it can be used in the form of micro- or nano-thin films. Using these films, mini or micro-structured devices can be manufactured through Micro-machining, microelectromechanical systems (MEMS), etc. MOS based chemi-resistive sensors react with the CO gas via reduction and oxidation, which affects the electrical properties of the sensing elements. It is this change in the electrical properties that helps to detect the presence of CO. The theory of CO gas sensing through adsorption processes in MOS substrates was first introduced in the sixties. However, it is in the last fifteen years or so that, some MOS materials were discovered to serve as excellent sensing materials for CO. Thus, they were used in industrial applications, the automotive industry (detection from vehicles), as well as indoor air quality monitoring. The main advantages of MOS based CO sensors include: (a) Small form factor; (b) simple detection and measurement system; (c) easy to fabricate; and (d) cost effective. However, there remain several challenges that need to be addressed. The most important challenge is related to the difficulty of sensing very low levels and constant occurrence of CO in air, which can affect human health severely. Hence, researchers started to look at alternative sensing approaches. These alternative solutions focus on the use of different materials for sensing at very low concentrations and with degradation of the sensing film and with greater stability. Recently, optical methods have opened a new door for detecting CO. Some researchers have used laser and infrared based detection using the light absorption principle [1,2,3,4,5,6,7,8,9,10,11,12,13,14,15,16,17,18,19,20,21,22].

Our goal is to provide a broad study on the research conducted on materials, methods, and their response to carbon monoxide, by which researchers can further improve sensing methods having low concentration detection ability, low cost and achievable operating conditions. In addition, we predict that sensing, measurement, and CO control systems can benefit from a direct control of the sensing mechanisms, as well as of the actuators. Such direct control can leverage the real-time interaction between the real physical world and the sensing and data computation components toward better decision making. We propose to employ a cyber-physical system (CPS) modeling and system development approach for such real-time controls. The CPS concept is relatively new and provides a comprehensive modeling framework to account for both the physical (real world, CO and other gases) and the cyber (sensors and their data, as well as all storage and data mining). We view this CPS approach as the integration of wireless sensing network (WSN) with embedded control techniques through which best decisions can be made towards controlling the physical component. High performance CO sensors will result in a high-performance CPS framework. Moreover, if CO sensors can be implemented with other household and commercial environment sensors in a CPS network, the results will be a fully controlled, real-time system [23,24,25,26,27,28,29,30,31,32,33,34,35,36,37,38,39]. To this end, the objective of this review paper are: (1) Introduce the necessity of sensing CO; (2) describe a CPS framework for CO sensing and control; (3) describe the mechanisms of CO detection through MOS based techniques and discuss recent studies on CO gas sensing based MOS techniques, focusing on sensitivity, response time, stability, doping, dependency on several parameters etc.; (4) describe recently proposed new optical detection methods; and (5) conclude with a summary that highlights several trends that we identified and projects that we make. We hope that this review article will help future research to specifically select the most appropriate material and sensing techniques for a given application domain, to improve the sensing, as well as the fabrication process towards the application in increasingly automated and real-time cyber-physical systems.

## 2. Necessity of Carbon Monoxide Detection

As a colorless, odorless, and tasteless gas, carbon monoxide is very harmful to human health as it can lead to death. Because humans cannot detect it directly, it is imperative to develop effective and cost-efficient smart sensing systems that can detect, and measure CO. Both natural and artificial sources can create carbon monoxide gas. The highest amount of exposure usually happens indoors, in places, such as garages, kitchens, etc. CO is generated during combustion that takes place in combustion engines, stoves, water heaters, generators, lanterns, and gas ranges or during burning charcoal and wood [21]. Acute exposure to carbon monoxide poisoning can result from any fossil fuel being burned. Long-term CO exposure can also result from constant exposure to automotive exhaust, smoke, and industrial sources (foundries, mills etc.) [19,20,21,22,23].

CO poisoning takes place through a mechanism that follows several chemical reactions. CO can produce carboxyhemoglobin via bonding with red blood cells (hemoglobin). This can happen due to its oxygen like chemical structure. The formed carboxyhemoglobin, in turn, obstructs the red blood cells to bind with oxygen. Hence, the transport of oxygen in the human body decreases, which results in the decreased levels of oxygen reaching the body tissues (histotoxic hypoxia). Consequently, common resultant health issues manifest as: Headache, nausea, vomiting, inertia, unconsciousness, weakness, hypotension, coma, inflammation of existing diseases, confusion, depression, hearing problems, etc. [19,20]. Often CO poisoning can be a cause for diabetes, parkinsonism, rhabdomyolysis, motion disorders and other severe health issues in children and pregnant women. A small amount of CO over a long period of time or a large amount of CO over a short period of time can kill a person within seconds to hours depending on the dose. The concentration of carbon monoxide is measured in parts per million (ppm) and parts per billion (ppb). High concentrations of CO can immediately affect vital organs and long-term exposure to low concentrations of CO can also create severe health issues (to children and to the fetuses of pregnant women). A summary of health problems caused by exposure to CO is presented in Table 1, where the problems are listed according to concentration and exposure time [19,20,21,22,23].

## 3. Cyber-Physical System Framework for CO Monitoring

**Figure 1:**
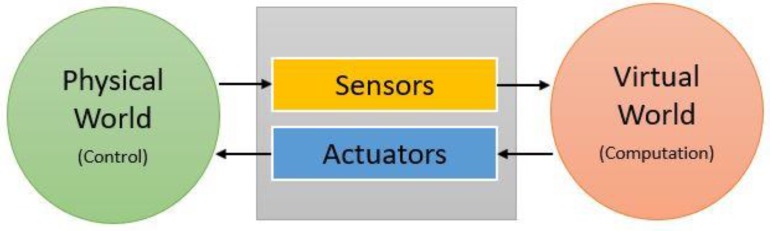
Basic cyber-physical system (CPS) structure.

The cyber-physical system (CPS) is a recent theoretical concept that marries the cyber world (computation) with the real physical world within one single modeling framework [24,25]. We propose to apply the CPS modeling approach to CO detection, measurement and control. In this context, the physical world is monitored for gases via sensing techniques that are linked to the cyber component responsible with computations, including storage and data analytics. Controls are deployed via actuators that receive control signals from the cyber algorithms on the cyber site. The actuator actions have immediate impact on the physical world. Currently, wireless embedded sensors and actuators systems are used in many application domains, including environmental and medical devices, autonomous vehicles, navigations and smart structures, where the CPS modeling and system optimization framework has been applied [24,25,26,27,28,29]. A simplified illustration of a basic CPS model is shown in Figure 1 that shows the interaction between the two main components via sensors and actuators.

Cyber-physical systems can be implemented in different types of environmental monitoring, such as air quality monitoring, greenhouse monitoring, humidity sensing, soil moisture sensing etc. In these CPS, the physical world can be controlled by sensing and monitoring any specific element through connected sensors, actuators and embedded devices. The sensing elements operate as the inputs that feed information to the cyber component. This information is supplied to computational systems that use it inside optimization algorithms for the purpose of making real or near-time decisions. These decisions in turn command the actuators that serve as output proxies to the physical world, thereby completing the loop illustrated in Figure 1. Because the computation is often done in the cloud, the CPS is connected to the internet (hence the terminology internet of things (IoT)) via WiFi or other wireless or wired communication technologies [30,31,32,33,34,35,36,37,38,39].

**Figure 2:**
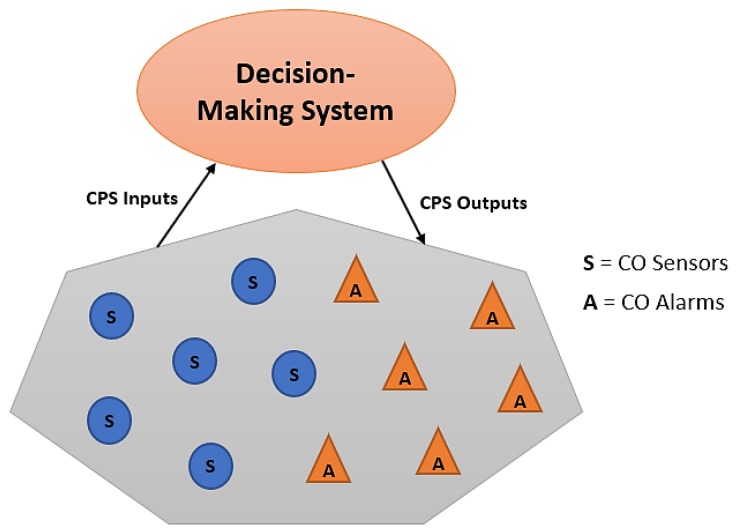
Example CPS based framework for carbon monoxide detection and control.

Our idea is to apply the CPS modeling framework to the problem of monitoring carbon monoxide gas in residential and industrial environments. Such a framework provides versatile common platform or ground for comparing existing CO monitoring approaches and for system design and optimization. Thus, CO gas sensors might be deployed in houses or industrial buildings to monitor CO concentration levels. When the CO concentration level crosses a pre-specified specific limit, alarms can be triggered. These events are recorded by the cyber computational component of the CPS, which is responsible with the generation of control commands to mitigate CO problem. The commands control the actuators that impact directly the plants that represent the main cause of CO gas generation. These commands are calculated and generated in real-time for maximum effectiveness. By following a distributed sensor network of Reference [33], Figure 2 shows a diagram of a candidate CPS approach that could implemented for CO monitoring and control.

**Figure 3:**
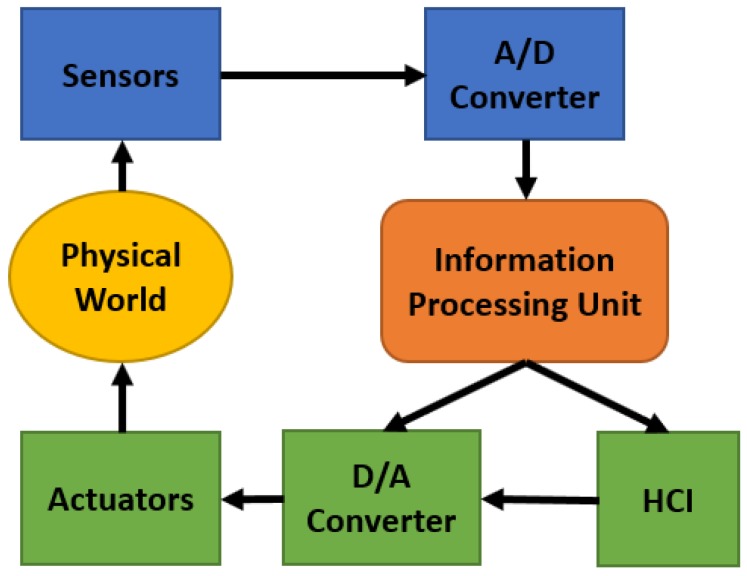
Components of a whole cyber physical system.

Generally, four types of cyber physical system have been proposed by the researchers. They are: (a) Reactive CPS; (b) Hybrid CPS; (c) Dedicated CPS; and (d) Dynamic CPS. Reactive CPS has a regular interaction with environment and performs at a speed maintained by environment. Hybrid CPS is a mixture of analog and digital parts. Dedicated CPS focuses on a fixed application having a minimum number of resources. Dynamic CPS has very frequent connection with environment, which has high amount of sensor data. For making a CPS system, besides sensors and actuators, A/D converter (analog to digital converter) and D/A converter (digital to analog converter) play big roles. A/D converter can pass the sensor data to the information processing system. Information processing system gives the processed data to the D/A system, which provides control to the actuators and HCI/HRI (human computer interaction or human robot interaction). Figure 3 shows the different components of CPS respectively [35,36,37,38]. Networking at multiple scale is also very important for making a useful cyber physical system. Such as in medical sector, if all the sensors along with CO sensors are connected in a single network, it can control all the sensing data at a time and provide immediate execution to all the actuators.

**Figure 4:**
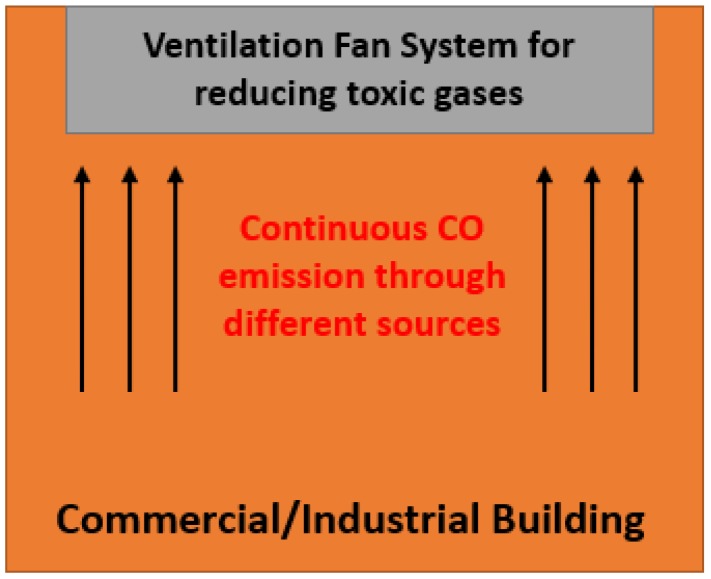
Commercial building ventilation system.

The main benefits of this IoT-enabled CPS-based approach of designing CO monitoring and control systems include: (1) Real-time and automated CO monitoring that directly affect personnel health; (2) energy savings and controlled ventilation; and (3) increase of productivity. In the industrial environment, CO is produced at a very high rate. That is why ventilation system runs all day long for properly ventilating the building to keep people healthy. Figure 4 shows the industrial building configuration. Reliable CO data is needed in order to control this ventilation system. CPS based CO sensing network can give real-time data. This reliable data can run the ventilation system at lower settings that can still maintain the proper ventilation. This controlled ventilation system ends up to huge energy savings. Moreover, in residential and industrial area, it is very difficult to work under very harsh condition having high temperature, high humidity and emission of CO. Thus, it is needed to sense and control all these things. By the sensors, we can only sense; but if all sensors can be implemented within a single CPS system, we can monitor every sensible item and control their sources (such as we can control light source and heating source at a time by monitoring temperature and CO through sensing network. This implementation can increase the productivity of any kind of work. Besides, through this CPS-based approach, one can easily monitor the air quality sensing the CO concentration. This has an immediate impact on the well-being of people living in their homes and working in factories. Continuous well-being has a long-term positive impact on workers. In addition, intelligent control through the CPS approach of the elements that generate CO or that help to control the CO levels (i.e., heaters, stoves, generators, ventilators etc.) can result in energy saving in household environment as well.

## 4. Metal Oxide Semiconductors Based CO Sensing

The most researched technology for CO detection is the metal-oxide semiconductor (MOS) solution. Researchers investigated MOS sensors for some time with special focus on carbon monoxide gas sensors with very high sensitivity, selectivity, responsibility (fast response/recovery time), adsorptivity (of oxygen), stability and reversibility, very low power consumption and low fabrication cost. The sensing process involves several steps or tasks. The first task of the metal-oxide sensing layer is to determine the CO gas incorporated with the electronic change of oxide layer surface. This determination of target gases is facilitated by the reaction between the adsorbed oxygen molecules and the CO gas molecules. The oxygen adsorption onto the oxide surface causes a variation of the $e^{-}$ flow (electrical conductivity). This results into a change in the resistance of the oxide surface layer [40,41,42]. From the measurement of this electrical property, the sensitivity of the oxide layer, due to CO gas is determined by comparison to the case of no presence of CO gas. The reactions that occur are described by the following expressions [43,44,45,46,47,48,49,50]:

(1) $$O_{2}↔O_{2}{}^{-}\;,$$

(2) $$O_{2}{}^{-}+e^{-}↔2O^{-}\;,$$

(3) $$O^{-}+e^{-}↔O^{2-}\;,$$

(4) $$2\mathrm{CO}+{\;O}_{2}{}^{-}\to 2{\mathrm{CO}}_{2}+e^{-}\;,$$

(5) $$\mathrm{CO}+{\;O}^{-}\to {\mathrm{CO}}_{2}+e^{-}\;,$$

(6) $$\mathrm{CO}+{\;O}^{2-}\to {\mathrm{CO}}_{2}+2e^{-}\;.$$

**Figure 5:**
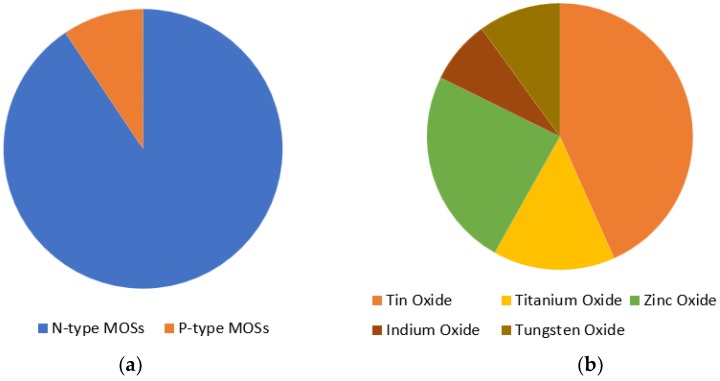
(**a**) Metal oxide semiconductors (MOSs); and (**b**) N-type MOSs, used for gas sensing.

**Figure 6:**
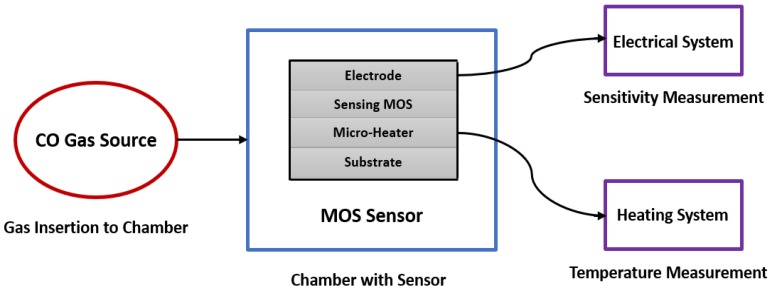
Basic block diagram of MOS thin film-based CO gas detection system.

The oxygen adsorption increases if the operating temperature increases (from $O_{2}{}^{-}$ to $O^{-}$ and $O^{2-}$). $O_{2}{}^{-}$ works at temperatures less than 150 °C, $O^{-}$ works at temperatures within 150 to 400 °C and $O^{2-}$ works at temperatures higher than 400 °C. However, temperature needs to be kept at an optimal value, due to its impact on the reliability and lifetime of the sensor. The adsorption also increases with the proper use of doping of the metal-oxide through which sensitivity can be increased. While both n-type and p-type metal oxide semiconductors are used for gas sensing, n-type is more popular. Therefore, the number of n-type MOS-based sensor is generally high. In Figure 5 (which is prepared from the data of Reference [50]), a brief statistic is given about the metal-oxide semiconductors, which are mainly used for gas sensing application. In general, MOS is used as both thin and thick film to detect CO gas. Thin film is mostly used in MEMS (micro-electro-mechanical systems) based gas sensors. The MOS thin film is combined with a micro-heater in order to create a high operating temperature, which enhances the sensor performance. Electrodes are needed for thin film resistance measurement, through which sensitivity is obtained. Sensitivity, response time and recovery time are three main performance characterization parameters. Response time is the time for responding to a step concentration change from 0 to 90% of the saturated value and recovery time is the time required for the sensor signal to return to 90% of the initial value) [40,41,42,43,44,45,46]. A basic block diagram of a MEMS metal oxide semiconductor thin film-based CO gas detection system is shown in Figure 6.

## 5. N-Type Metal Oxides for CO Sensing

All sensitive metal oxide semiconductors react with CO gas by adsorbing oxygen. However, the variation in electron flow (changes in conductivity) is different in n-type and p-type MOS. When n-type MOSs is exposed to CO gas, the gas is oxidized, and the resistance of the sensing film decreases. This happens because the resistive core and semiconducting shell works in series in n-type metal oxides. Therefore, when oxidization takes place, the residual electrons are inserted into the semiconducting core, which results in an increase in sensor conductivity [47,48,49,50]. The most used n-type metal oxide semiconductors for CO gas sensing are Tin Oxide (SnO_2_), Titanium Oxide (TiO_2_) and Zinc Oxide (ZnO). Indium Oxides (In_2_O_3_ and In_3_O_4_), Cerium Oxide (CeO_2_) and Tungsten Oxide (WO_3_) have also been investigated recently [47,48,49,50,51].

### 5.1. Tin Oxide (SnO_2_)

Tin oxide (SnO_2_) is the mostly utilized n-type metal oxide semiconductor, because it provides great sensitivity in the case of carbon monoxide sensing. However, different types of SnO_2_ (undoped and doped thin film, nanowire, nanoparticle, nanocluster, etc.) were investigated as well. For example, Kolmakov et al. [52] investigated on SnO_2_ nanowires and found that the smallest optimum diameter was around 60 nm for the adsorption of oxygen. They prepared n-type SnO_2_ nanowires from p-type SnO sequentially. The authors evaluated the I–V characteristic for different temperature values in a CO environment and found that the conductance was increasing, while the response time remained constant at around 35 s. They worked under 200, 250 and 280 °C; but they did not mention about the optimum temperature. Du et al. [53] studied the dependency of sensitivity on the thickness of SnO_2_ thin film. They fabricated SnO_2_ thin films with different thicknesses (within 1.59 nm to 5.87 nm) using the atomic layer deposition (ALD) method and measured the responses to CO for temperatures in the range of 200 to 325 °C. In the deposition process, SnCl_4_ and H_2_O_2_ were used as reactants and in the sensor construction, Au, Pt, and Pt were used as electrode, heater and contact wire respectively. They monitored the sensor resistance as sensor response under O_2_ and CO exposure using the following equation: R_O2_/(R_O2_ + R_CO_). The best response was found at 2.62 nm thickness at 300 and 325 °C. The response was increasing up to this thickness, but, it drastically fell after that. There was no decrease of resistance at thicknesses greater than 2.62 nm. Response time was also shorter at increased temperatures and the highest was found within 250 to 325 °C. 

**Figure 7:**
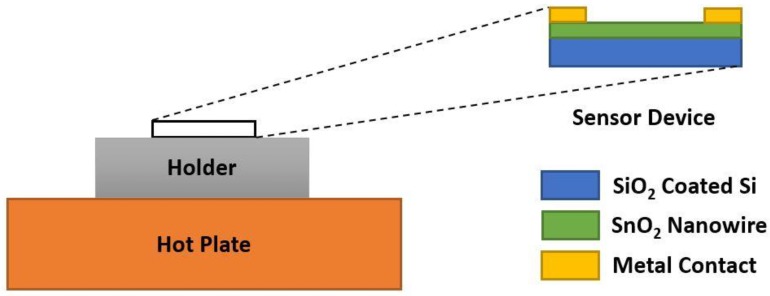
Fabricated SnO_2_ nanowire sensor device structure.

Tischner et al. [54] studied SnO_2_ thin films with thicknesses of 50–100 nm deposited on SiO_2_/Si substrates. They used the spray pyrolysis method under very high temperature (using hotplate of 450 °C). CO gas was mixed with synthetic air and DC currents of 200 µA were applied and then the DC voltage drop was measured, which was then used to find resistance (response measurement). They found good sensor response to 4 ppm CO at 350 °C and at decrease of resistance for 0–200 ppm CO, which proved its low concentration detection ability. The sensor response decreased in humid air which also indicated its humidity dependency. Köck et al. [55] investigated on SnO_2_ nanowire fabricated via the spray pyrolysis method at atmospheric pressure for detecting CO and CH_4_ together. Titanium (Ti) or Gold (Au) contact pads were used at both ends, as shown in Figure 7. Several tin oxide nanowires with diameter within 30–400 nm responded well at low ppm CO (4 ppm at 250 °C) with a moderately fast response time (around 25 s). However, as the sensor resistance was increased, strange behaviors were discovered, due to ionosorbed oxygen. This strange behavior stopped after 300 °C, which indicated the necessity of higher operating temperature on oxygen adsorption for this sensor to work properly.

For reformer and membrane fuel cell applications, Lee et al. [56] investigated on a MEMS based CO gas sensor using SnO_2_ thin film. Stainless steel foil and aluminum nitride were used as substrate and isolation layer respectively in this micro-machined device. They deposited 100–300 nm SnO_2_ thin film through the RF sputtering. Measurements of sensor response were performed at 100, 300 and 1000 ppm CO gas at temperatures within 100–350 °C. They found that the sensitivity increased up to 300 °C and was highest for the smallest thickness (100 nm) of thin film. AFM (atomic force microscopy) and SEM (scanning electron microscopy) micro-images proved that smaller grains of 100 nm thin film provided higher oxygen adsorbtion, which in turned enhanced the sensor sensitivity. Both the response time and recovery time were shorter for 100 nm than for 200 and 300 nm films. The optimum temperature was found to be 270 °C for 1000 ppm CO gas and the constant sensitivity, (R_a_ − R_s_)/R_a_, was found at around 59% for 100 nm where the reproducibility and stability of this sensor was proved. They also observed the annealing effect on the sensor and that the sensitivity was higher for unannealed films (films without heat treatment). Pt and Au sputtering effect on sensor response were also studied and found that lower sputtering time resulted in higher response at all operating temperatures.

#### 5.1.1. Copper Doped Tin Oxide

**Figure 8:**
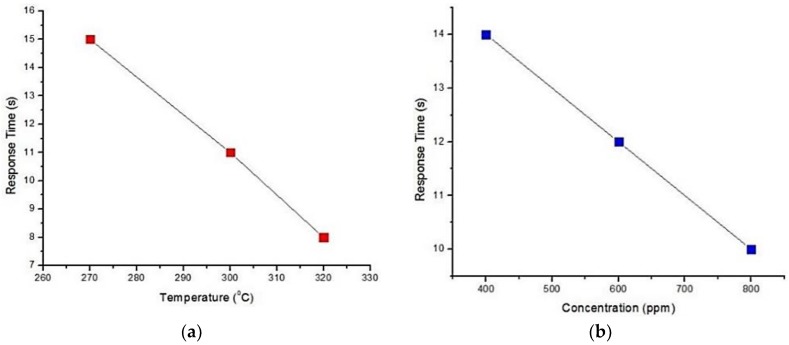
Response time of SnO_2_-Cu/Pt—(**a**) at different operating temperature to 1000 ppm CO gas; and (**b**) to different concentrations of CO gas at 250 °C operating temperature.

Sharma et al. [57] investigated the sensitivity, selectivity and stability of Copper (Cu) doped SnO_2_ thin films for sensing CO gas in integrated gas sensor array applications. They studied two thin films: (1) Cu doped tin oxide with Platinum on top (SnO_2_-Cu/Pt) and (2) Cu doped tin oxide with Platinum and SiO_2_ layers on top respectively (SnO_2_-Cu/Pt/SiO_2_). In this process, Cu doping concentration was 0.16 wt.% and thin film thicknesses for doped SnO_2_, Pt and SiO_2_ are 3000 Å, 10 Å and 100 Å respectively. The thin film was deposited at room temperature using RF sputtering. The sensitivity of SnO_2_-Cu/Pt thin film was measured via electrical response time observation, i.e., voltage change per unit time (with and without gas). The voltage change was measured within 5–10 s, when 400, 600, 800 and 1000 ppm concentrations of CO gas were supplied at 250, 270, 300 and 320 °C. It was found that the voltage change was faster and larger for 1000 ppm at 320 °C, which indicated that higher operating temperature and higher gas concentration can help to reduce the response time. This is illustrated in Figure 8. This can be explained by the adsorption of CO gas molecules that increases at high operating temperature, which makes the diffusion faster.

Stability of SnO_2_-Cu/Pt thin film was measured at 1000 ppm CO concentration and 300 °C and compared to undoped tin oxide thin film from. It was found that SnO_2_-Cu/Pt thin film (annealed at 450 °C for 30 min) was stable after 300 h. Any kind of design or structural errors, phase shift of dopant materials (Cu/Pt) in sensing film and variation in the surrounding environment can be the reasons for taking long time to become stable. It was suggested that annealing temperature and time could be optimized for enhancing the thin film stability, as well as electrical properties. Moreover, when 1000 ppm CO and H_2_ gases was given to SnO_2_-Cu/Pt/SiO_2_ thin film at 270 °C and 320 °C, it was found that this thin film was not responsive to CO gas, which proved the selectivity of SnO_2_-Cu/Pt thin film to CO gas [57].

#### 5.1.2. Palladium and Platinum Doped Tin Oxide

**Figure 9:**
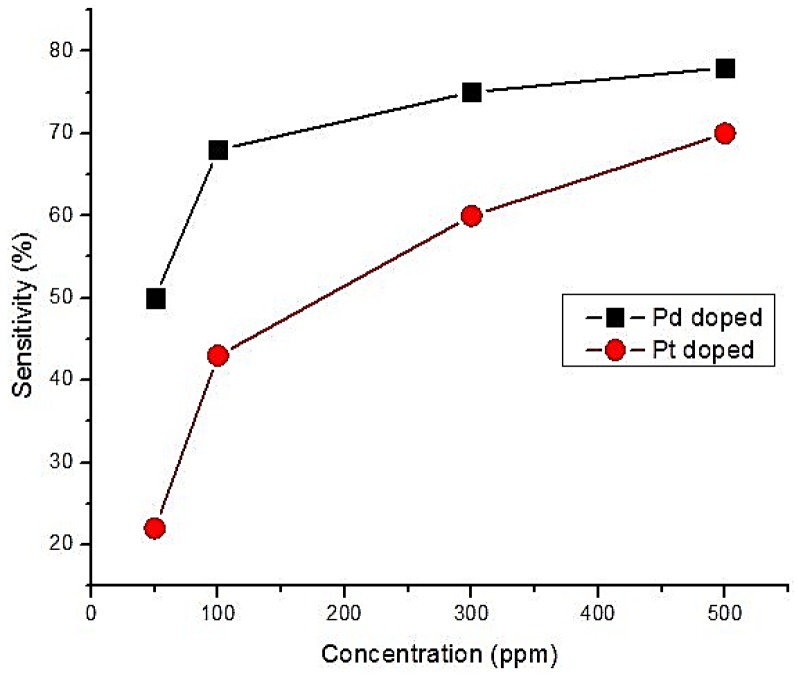
Sensitivity of 2% Pd-doped and 2% Pt-doped Tin Oxide thin film according to different concentrations of CO gas.

Sensitivity of CO gas to Palladium (Pd) and Platinum (Pt) doped SnO_2_ nano thin films in dry air was studied by Menini et al. [58]. The authors used polysilicon as heater on top of the silicon membrane. The Pd or Pt doped thin film was deposited on top of the heater. Doping concentration was 2% for both doped thin films. The heater was used to increase the operating temperature to 450 °C. They reported the sensitivity equation as following:

Sensitivity = [(R_g_ − R_a_)/R_a_] × 100% (7)

where, R_g_ and R_a_ are the resistance of sensing thin film with and without CO gas respectively. It was reported that Pd doped SnO_2_ thin film was more sensitive than Pt doped SnO_2_ in dry air. This is illustrated in Figure 9. The duration between recovery time and response time was lower for Pd (around 50 min) than Pt (around 60–65 min).

**Figure 10:**
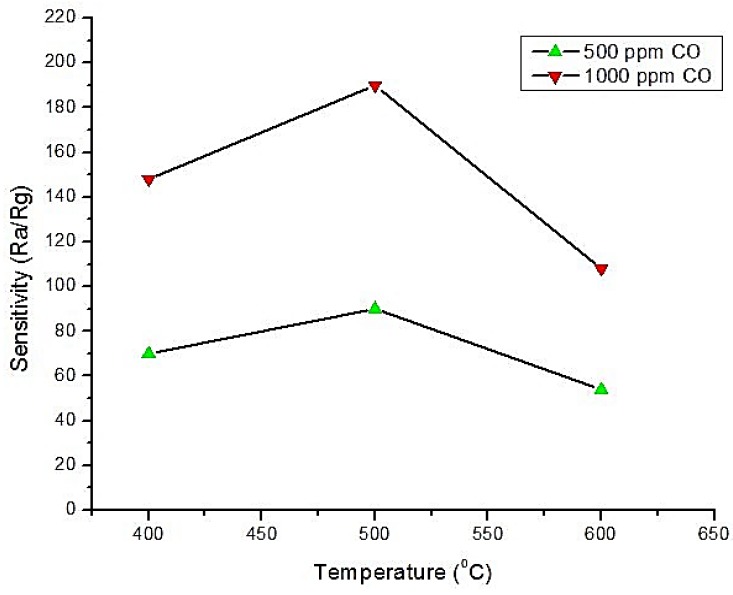
Effect of calcination (in N_2_) temperature on Pt-SnO_2_ to 500 and 1000 ppm CO gas.

Pd-SnO_2_ and Pt-SnO_2_ CO gas sensors were investigated in Kim et al. [59] and Wang et al. [60] at very low temperature (60–65 °C), i.e., room temperature gas detection. The authors of Reference [59] used 1.5 wt.% Pd doping and several mixtures of HPC (Hydroxypropyl Cellulose) for improving the sensitivity. From the measurement of the effect of HPC on sensors, it was found that 15% HPC mixing with 85% Pd-SnO_2_ showed the maximum response towards 1–30 ppm CO gas concentration. Other ratios had very weak response. It was reported that the relative humidity (%RH) had no effect on this sensor in both dry and wet air at 60 °C. Furthermore, it was found that this technique offered a very good repeatability (almost 5–9.3%) and that the relative response was linear with the concentration. The relative response for 18 ppm CO gas was almost constant over 12 runs and 20 days, which demonstrated a good stability. In the study from Reference [60], Pt-SnO_2_ porous nanosolid was fabricated and calcined in both nitrogen and air. It was reported that the sensor calcined in N_2_ showed excellent response while the sensor calcined in air had no response towards CO gas (0 to 1000 ppm). The effect of calcining in N_2_ at different temperatures was studied and it was discovered that calcination at 500 °C offered the highest sensor response towards every gas ppm level illustrated in Figure 10. Another finding was that this sensor response was decreasing, almost linearly, according to the increase in the sensor thickness and in the relative humidity in air. These findings confirmed that the efficacy and selectivity of the fabricated sensor were some of the best.

Yuasa et al. [61] developed another palladium oxide (PdO) doped with SnO_2_ nanoparticles sensor using Pd(OH)_2_ and Sn(OH)_4_. They used the reverse micelle method and found that the size of PdO nanoparticles had a good impact on the sensing performance when it was used as a dopant on tin oxide. The sensor response measurement was done at 300 °C for 200 ppm gas concentration where the optimum Pd doping was determined as 0.1 mol% (highest response was around 320–325). It was shown that the optimal use of PdO loading on SnO_2_ can improve the sensing properties drastically.

#### 5.1.3. Gold Doped Tin Oxide

**Figure 11:**
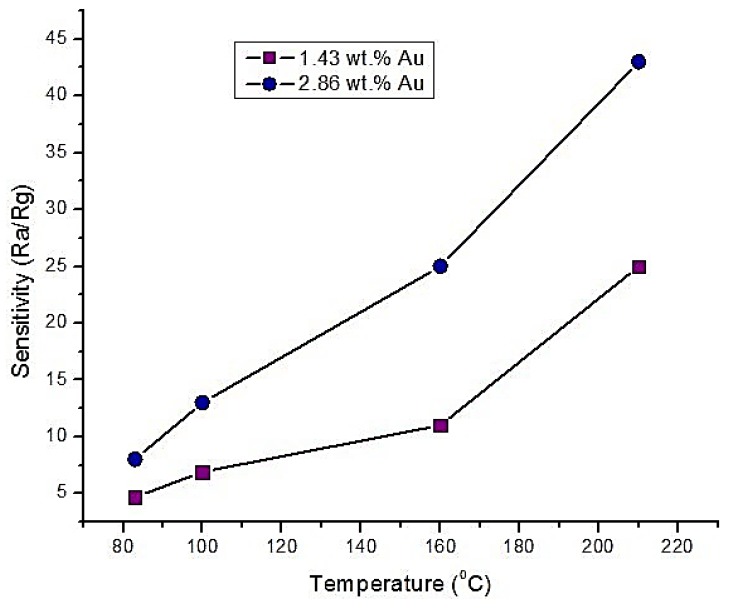
Sensitivity of two different Au doped SnO_2_ thick films at different temperatures.

In [62], a Gold (Au) doped SnO_2_ thick film sensor was fabricated for sensing CO gas at different doping weight percentages. The fabricated film used 10 different Au/SnO_2_ powders (within 0.36 wt.% to 3.57 wt.%) via the deposition-precipitation method. The films were investigated at four different temperatures (83, 100, 160 and 210 °C). The authors evaluated the sensor response using the expression R_a_/R_g_ (where R_a_ is the resistance of the film in air and R_g_ is the resistance of the film in CO gas) at different gas concentrations (0 to 4000 ppm). They found that response of the film increased with the increase in CO gas concentration and the increase of the operating temperature (as shown in Figure 11). It was reported that the highest response was found at 2.86 wt.% doping. Higher doping concentration that were higher or lower than this optimum value (2.86 wt.%) resulted in lower responses. The response and recovery times were studied too, and it was found that they decreased with the increase in the operating temperatures and gas concentrations. For example, they found the difference between the response and recovery times as 16, 7, 4 and 1 s at the above four different temperatures and for a gas concentration of 500 ppm.

The study in Reference [63] used successive ionic layer deposition to deposit Au doped SnO_2_ nanocomposites on substrate. The fabricated films provided enhanced CO sensing abilities. Sensitivity measurements were done for concentrations of 2000 ppm and without gases at temperatures of 450 °C for different deposition cycle numbers. There was found that the sensitivity increased with the increase of the number of deposition cycles. It was also found that when temperature increased from 100 °C to 450 °C, the CO conversion (oxidation) rate increased significantly.

**Figure 12:**
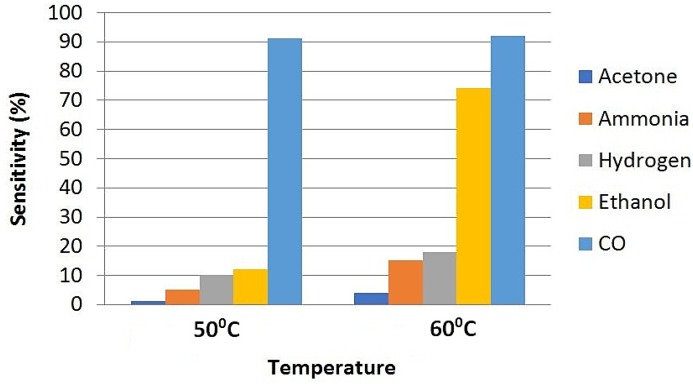
Selectivity of Au/SnO_2_ for CO at 50 °C and 60 °C.

Carbon monoxide sensing at room temperature was researched by Manjula et al. [64]. The authors fabricated hydrothermally synthesized Au/SnO_2_ to achieve better sensitivity and selectivity in sensing of CO gas. The sensor response was evaluated via resistance measurements at varying temperatures and for different doping concentrations (0.5 to 2 wt.%). They found that highest response for 1.5 wt.% Au. Moreover, the sensor response of 1.5 wt.% Au/SnO_2_ was analyzed for different CO gas concentrations (up to 500 ppm) and different relative humidity (55% and 70%) values. It was found that the response was very fast and stable for 1.5 wt.% at room temperature for 500 ppm CO gas. However, no effect of humidity on this sensor response was found. The selectivity of this sensor was investigated for CO along with other gases as illustrated in Figure 12, where all the gases had 10% concentration in the air.

#### 5.1.4. Multiwalled Carbon Nanotube Doped Tin Oxide

Zhao et al. [65] studied multiwalled carbon nanotube doped SnO_2_ nano-particles (MWCNT/SnO_2_) for sensing the CO gas. They used 0.1 wt.% doping concentration and reported higher crystallinity at 400 °C calcination temperature. They measured the resistance of the pure and hybrid oxide nano-materials in both gas and non-gas conditions, through which they determined the sensitivity to CO gas. The initial resistances were 4.6 MΩ, 0 and 1.3 KΩ, respectively, and the sensitivity to 100 ppm CO gas was 15%, 0 (almost) and 46% for SnO_2_, MWCNT and MWCNT/SnO_2_ respectively. They also measured the response and recovery times through resistance-time curves at 300 °C. They found that MWCNT/SnO_2_ could offer almost three-times higher sensitivity and significantly shorter response time, recovery time, and better stability compared to undoped SnO_2_.

Another study on MWCNT doped SnO_2_ was done by Leghrib et al. [66]. They investigated the sensitivity obtained by sensors constructed with different MWCNT and SnO_2_ ratios for low ppm levels of CO at low operating temperatures (room temperatures and 150 °C). The response time was significantly higher than that of pure nanomaterials. However, a lower resistance at room temperature contradicted the results from previous work. It is also found that MWCNT-SnO_2_ was very at low CO concentrations 2, 5, 10, and 20 ppm. Furthermore, the responsiveness reduced at lower doping concentrations and high levels of moisture.

#### 5.1.5. Vanadium Doped Tin Oxide

**Figure 13:**
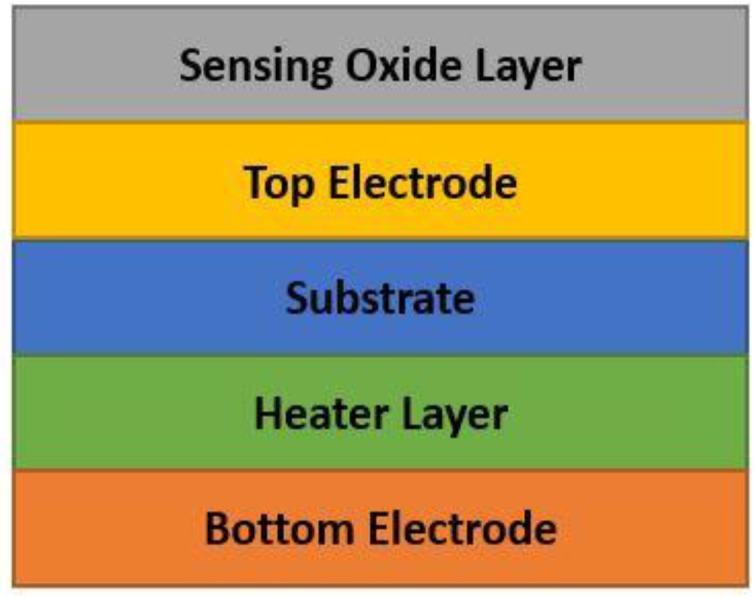
Device structure for V/SnO_2_ CO gas sensor.

Wang et al. [67] researched on vanadium (V) doped SnO_2_ gas sensors and investigated the role of V doping on the sensitivity of tin oxide. The operation of this sensor follows two main mechanisms: (1) Oxygen activation-CO oxidation and (2) Adsorption-Desorption where both depend on the role of vanadium redox pairs and oxygen vacancies. Researchers fabricated the V-SnO_2_ nanoparticles using the co-precipitation method and characterized the crystallinity through XRD (X-ray diffraction) and FTIR (Fourier-transform infrared spectroscopy) techniques. They found that greater vanadium doping made the sensor less crystalline. They used Au electrodes and RuO_2_ heater during measurements (for a layered structure, shown in Figure 13). Experiments showed that resistance increased with the increase of oxygen concentration, which demonstrated the oxygen adsorption and n-type behavior of V-SnO_2_ sensors. The sensor response (R_a_/R_g_) according to time, temperature and concentration were reported for five different doping ratios (V/Sn = 0, 0.05, 0.1, 0.15, 0.2). The sensor with 0.05 V/Sn ratio had the highest sensitivity towards CO gas at 175 °C, which was identified as an optimum ratio in this approach. Stability experiments, conducted over a period of 72 h continuously, indicated that V-SnO_2_ sensor was very stable. 

#### 5.1.6. Calcium Doped Tin Oxide

Ghosh et al. [68] introduced calcium (Ca) doped SnO_2_ sensors for fast detection of CO at low pp levels. 5 wt.% and 10 wt.% Ca doped SnO_2_ was fabricated via the sono-chemical method. The sensor response (R_a_/R_g_) was characterized at 350 °C within a lower CO concentration region (1–30 ppm) where the response was linear with the increase in the CO gas concentration. The highest response was achieved at 30 ppm. Besides, the response to 30 ppm CO was stronger at high temperatures. The authors measured the resistance for 1 ppm CO from and found the difference between response and recovery times was 25–28 s. The optimum of Ca doping was found as 5 wt.% at 350 °C, which showed highest response and best efficiency compared to other doping concentrations of Ca and other metals (Au, Pt and Pd) on SnO_2_. This sensor displayed good selectivity compared to Ethanol and Methane at 350 °C and showed stability for very longer period (almost 1.5–1.8 years).

#### 5.1.7. Zinc Oxide Doped Tin Oxide

Nikan et al. [69] explored the use of zinc oxide (ZnO) doped SnO_2_ for CO sensing. They fabricated ZnO-SnO_2_ fibers (0.5, 1, 1.5, 2 and 5 wt.% doping concentrations) via the electrospinning method and measured the sensitivity towards CO gas. The calcination of ZnO-SnO_2_ sample was done at 450 °C for 3 h. It was found that at concentrations of 300 ppm, the optimum doping was 1 wt.% Zn, which showed higher response than other doping percentages at almost every investigated temperature (highest was at 300 °C). Furthermore, CO conversion (oxidation percentage) versus temperature was studied for 1 wt.% Zn doped and undoped SnO_2_ and it was found that CO conversion needed relatively low operating temperatures for pure tin oxide. The difference between response and recovery times for 1 wt.% Zn-SnO_2_ at 300 °C operating temperature was found to be 11 min.

#### 5.1.8. Copper Oxide Doped Tin Oxide

The authors of Jeun et al. [70] investigated CuO-SnO_2_ nano-hybrid foam-like sensors for various toxic gases, including carbon monoxide. Fabrication was done via electrothermal method of Sn-Cu and transfiguration of Sn-Cu to CuO-SnO_2_ via annealing at 700 °C. They found the sensitivity value to be 1.2 and the response time to be 6 min 30 s for 20 ppm CO gas at 250 °C. Another study on electro-spun CuO-SnO_2_ nanocomposite was conducted in Bai et al. [71] for annealing temperature of 600 °C. They measured the response over pure CuO and SnO_2_. At low level CO concentration (10 ppm), the heterojunction structured CuO-SnO_2_ performed better (maximum sensitivity, R_a_/R_g_ = 95) than pure oxides for all doping concentrations of CuO at any temperature (optimum doping and temperature was found at 30 wt.% and 235 °C). Moreover, the response of 30 wt.% CuO-SnO_2_ showed highest response at 235 °C compared to all other gases (ethanol, methanol, toluene, acetone and formaldehyde), which proved its excellent selectivity towards CO. In addition, it was found that the response was better at upper CO concentration levels (sensitivity = 150 at 30 ppm).

#### 5.1.9. Tin Oxide with Graphene and Silver Nanoparticles

**Figure 14:**
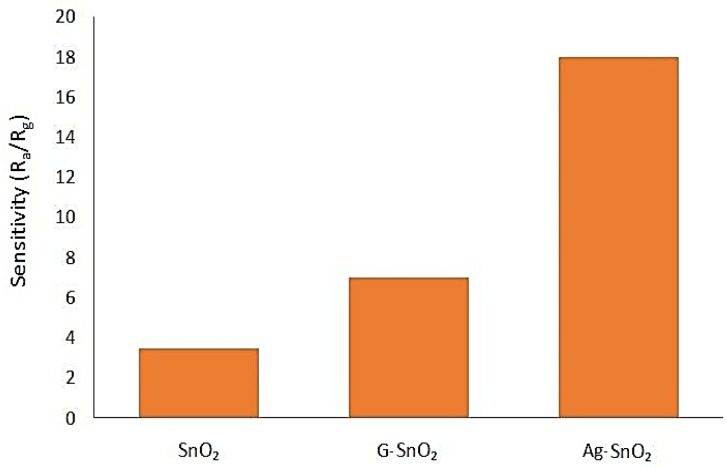
Sensitivity Comparison among SnO_2_, G-SnO_2_ and Ag-SnO_2_.

Kim et al. [72] explored improvements of photochemically fabricated SnO_2_ thin film by using graphene (G) and silver (Ag) nanoparticles. After annealing at 500 °C, SnO_2_ thin films were prepared with 0.0055 wt.% and 0.05 wt.% nanoparticle concentrations of Ag and graphene, respectively. The resistance analysis showed that these sensors worked like n-type semiconductors. The sensitivity at 100 ppm and 400 °C in dry air was significantly improved after incorporating these nanoparticles. This is shown in Figure 14.

#### 5.1.10. Summary of the SnO_2_ Based CO Sensors

In this section, we present Table 2 having summary of the most effective SnO_2_ based CO sensing techniques discussed in this review paper. This table shows their sensitivity and responses, optimum operating conditions, advantages and limitations.

### 5.2. Titanium Oxide (TiO_2_)

**Figure 15:**
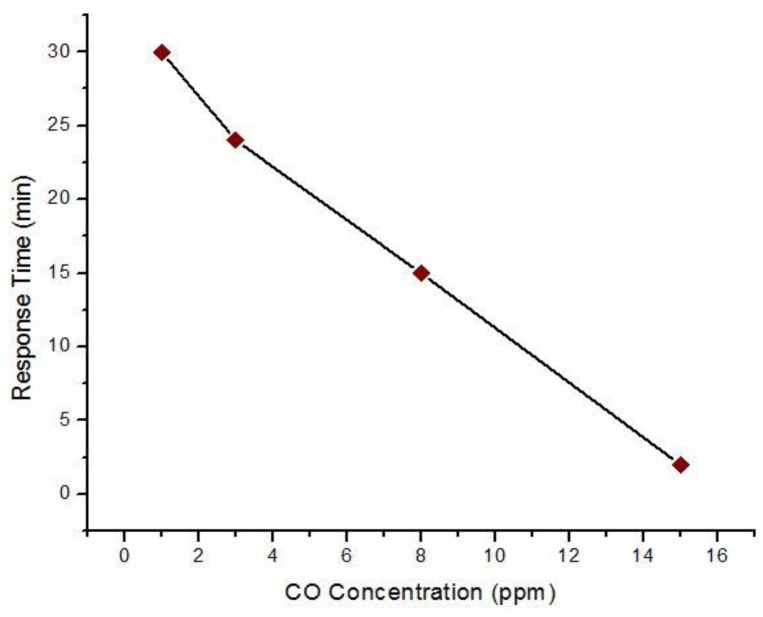
Response time of TiO_2_ (calcination temperature 600 °C) to 1–15 ppm CO gas at 200 °C.

Titanium oxide (TiO_2_) is another n-type metal oxide semiconductor, which was found to provide better sensitivity to CO gas. Park et al. [73] processed TiO_2_ nanofibers via the electro-spinning method. These nanofibers were with Polyvinyl Pyrrolidone (PVP). The sensors used Pt electrodes. SEM images showed that the radius of the fabricated nanofibers varied in the range 175–250 nm. The sensitivity was measured as a function of the calcination temperature. Higher calcination temperatures resulted in higher grain sizes. The optimum calcination temperature was found to be 600 °C, which offered the highest response (R_a_/R_g_ = 4.5) at 200 °C operating temperature for 25 ppm CO. Measurements for 1–15 ppm CO (see Figure 15) demonstrated that this type of sensor was very effective at very lower CO concentrations as well. Moderately low operating temperature and low concentration detection ability was found because of the high density of adsorption sites of nanocrystalline TiO_2_ nanofibers.

Moon et al. [74] fabricated NTHH (nanostructured TiO_2_ hollow hemisphere) based CO gas sensors via the deposition at room temperature and at high temperature calcination. They found almost 20 times higher percentage of resistance change at 250 °C for 1–500 ppm CO gas compared to plain TiO_2_ thin film. Comparison with results from Guidi et al. [75], Mohammadi et al. [76], Seo et al. [77] and Landau et al. [78] showed better response. SEM images showed that these NTHH films had a little space between hemispheres, which helped in the gas diffusion process. In dry air, the resistance change remained stable for 7 days, indicating a very good stability. Lee et al. [79] investigated how the porosity of TiO_2_ xerogel nano-thin film affects CO gas sensing properties. They performed SEM, XRD and FTIR to study the structural and morphological properties, including particle size, pore size, and anatase phase creation. It was found that the sensitivity and recovery time increased while the response time decreased with the increase of porosity percentage. Moreover, adsorption-desorption was also dependent on pore diameter (nm). The highest sensitivity, recovery and lowest response times were found for the thin film with the highest (62%) porosity and lowest pore size (3.3 nm).

**Figure 16:**
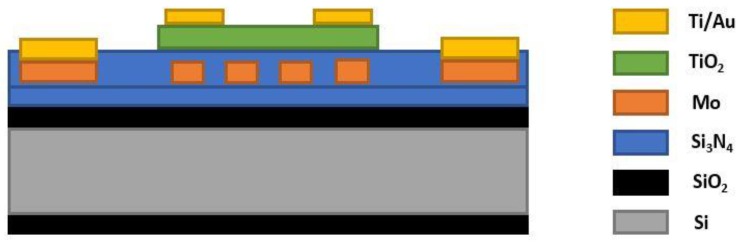
MEMS micro gas sensor device cross-section before etching.

Rao et al. [80] introduced miniaturized MEMS (micro-electro-mechanical-systems) based CO gas sensors using TiO_2_ thin film and Molybdenum (Mo) micro-heater. Molybdenum has a high melting point and a low thermal expansion co-efficient, hence, the authors fabricated a Mo based double spiral micro-heater whose temperature was varied through a Joule heating process. Silicon nitride was deposited as an isolation between sensing TiO_2_ thin film and Mo micro-heater, as illustrated in Figure 16. The authors reported that the Mo micro-heater could reach 800 °C at a low power consumption (104 mW) and a temperature gradient 9.5 °C. This offered a great improvement in CO gas sensing. Gas sensitivity measurements showed that after reaching an optimum 500 °C operating temperature, the MEMS sensor provided the highest sensitivity (84%) for every concentration of CO gas (1000–5000 ppb).

#### Multiwalled Carbon Nanotube Doped TiO_2_ thin film

Following the work of Reference [79], Lee et al. [81] tested TiO_2_ xerogel thin film integrated with 0.01 wt.% multiwalled carbon nanotubes (MWCNT) for CO gas sensing. The sensor fabrication was done via the sol-gel method. They reported that the sensitivity for MWCNT doped TiO_2_ film was 15.8. This was 7 times higher than that of pure film. The response for 50 ppm CO at 300 °C was also very fast (4 s). Moreover, sensitivity of this hybrid film showed good stability. In another study on MWCNT doped TiO_2_ thin film, Kim et al. [82] investigated the MWCNT doping effect on pure films. They found similar results as previous study.

### 5.3. Zinc Oxide (ZnO)

Another CO sensitive metal oxide semiconductor is zinc oxide (ZnO). ZnO is generally sensitive to many gases. That is why ZnO doped with different dopant materials has been used in CO gas applications. Doping improves its selectivity for different amount of CO gas concentrations at different conditions.

#### 5.3.1. Aluminum Doped Zinc Oxide

**Figure 17:**
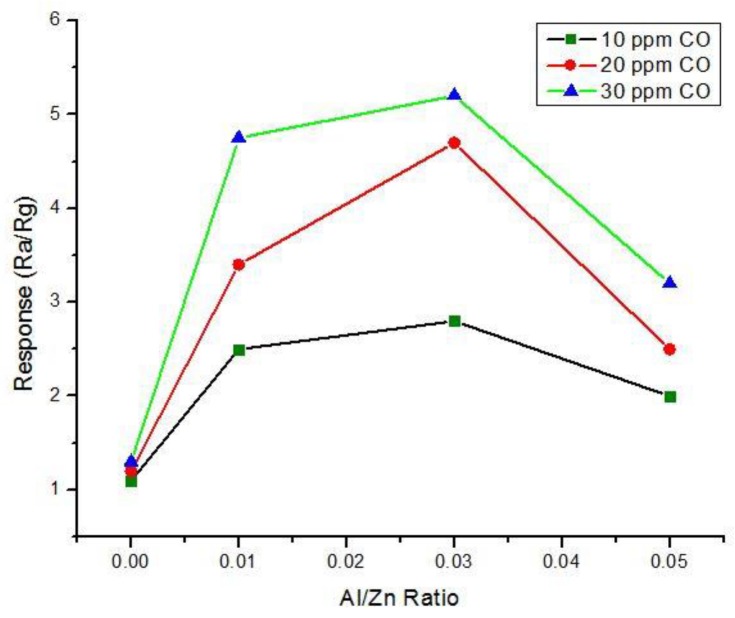
Response according to Al/Zn ratio towards 10, 20 and 50 ppm CO gas at 300 °C.

Chang et al. [83] investigated Aluminum (Al) doped ZnO thin films. 2 wt.% Al:ZnO (AZO) films having four different thicknesses (65, 188.5, 280 and 390 nm) were prepared via the RF sputtering process on SiO_2_/Si substrates. They found that the sensitivity of this sensor for 1000 ppm CO was decreased with the increase of its thickness and the optimum operating temperature was to be 400 °C. The response time was found to be very fast for low thickness values. In another recent research on AZO based CO sensor, Hjiri et al. [84] observed that Al doped ZnO nanoparticles displayed very high sensitivity and fast response to lower ppm of CO concentrations. They constructed AZO nanoparticles via the sol-gel method using four different Al/Zn ratios (0, 0.01, 0.03 and 0.05) for getting the optimum doping concentration. The authors reported that the resistance decreased with decreasing Al concentration. At 300 °C, the response of Al/Zn-0.03 was better than in for other ratios at 0–80 ppm CO gas concentrations (see Figure 17). At low temperatures <300 °C, Al/Zn-0.01 provided the better results. Al/Zn-0.03 also offered very fast and higher response compared to NO_2_.

#### 5.3.2. Copper, Palladium, Indium and Gallium Doped Zinc Oxide

**Figure 18:**
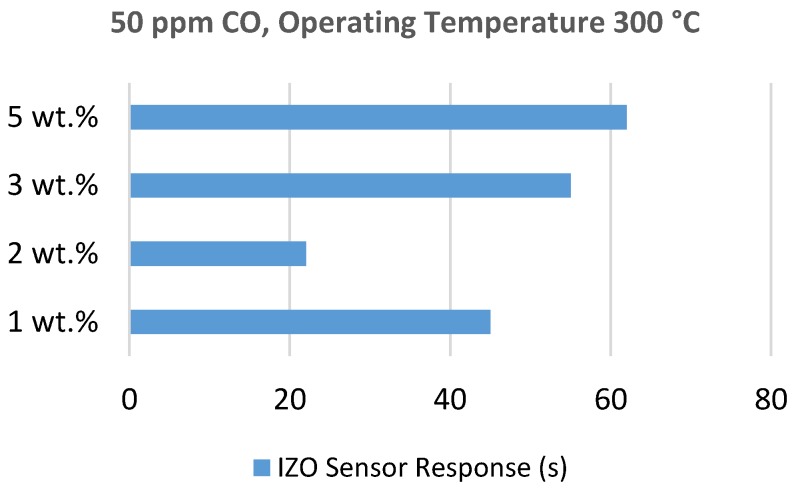
Response time for different in doping concentrations. IZO, Indium doped ZnO.

Gong et al. [85] fabricated Cu doped ZnO (CZO) nano-thin films (Cu/Zn ratio = 0.38) via the co-sputtering method and compared the response to pure ZnO films. The authors observed that the resistance decreased for different concentrations of CO at 150 °C, which proved this sensor’s sensitivity. For 20 ppm CO levels, the highest response was found at 350 °C. They explained the temperature dependency using the oxidation reaction described by Equations 1–6. Wei et al. [86] developed electrospun Pd doped ZnO (PZO) nanofibers with 35–80 nm radius range. These nanofibers showed moderate and better sensitivities at low concentrations of CO (1–20 ppm). They found the optimum temperature to be 220 °C for PZO, which was lower than for pure ZnO. To study the selectivity, they performed response measurements for CO, toluene, acetone, NO_2_ and CH_4_ and the response for CO was almost 5–6 times higher than for the others. In another study, Dhahri et al. [87] fabricated Indium (In) doped ZnO (IZO) nano-particles via the sol-gel method with different In atomic doping concentrations (0, 1, 2, 3 and 5 at.%). Towards 50 ppm CO, they found higher sensitivity for 1 and 2 at.% IZO nano-particles than pure ZnO. The optimum operating temperature was around 300 °C. IZO with 1 and 2 at.% had almost similar response to 0–80 ppm CO at 300 °C. IZO (2 at.%) had the faster response and recovery time than other doping concentrations (shown in Figure 18). Kim et al. [88] successfully tested Gallium doped ZnO nanowires (GZONW) using Ga doping within 0–5 wt.%. The optimum doping concentration they got was 3 wt.%. They found a significant resistance change for GZONW compared to pure ZnO at both room temperature and 200 °C.

#### 5.3.3. Cerium Oxide Doped Zinc Oxide

Kuhaili et al. [89] fabricated a mixed oxide-based CO gas sensor, where both Cerium Oxide (CeO_2_) and ZnO were deposited on the silica and alumina as substrate (for optical measurements and sensing measurements respectively). Platinum heater was attached to the backside of the substrate and platinum electrodes were integrated to sensing film. They reported the highest sensitivity of CeO_2_-ZnO to 10,000 ppm CO levels at an optimum temperature of 380 °C. The response and recovery time were determined as 44 and 40 s, respectively. However, this sensor was only examined at high concentration of CO.

### 5.4. Indium Oxide (In_2_O_3_)

Indium oxide is another promising n-type metal oxide semiconductor for CO gas sensing. There are two types of indium oxide: Indium (II) Oxide (In_2_O_3_), and Indium (III) Oxide (In_3_O_4_). In_2_O_3_ was investigated in Reference [90], where In_2_O_3_ microspheres were prepared and tested its sensitivity at CO concentrations of 10–50 ppm CO and at operating temperature of 400 °C. Sensitivity for hierarchical and hollow structures were found to be 2.16–3.81 and 1.99–2.79, respectively. The response time for both were 4–8 s and 5–10 s, respectively. Lim et al. [91] fabricated electro-spun In_2_O_3_ nanofibers (INF) and investigated the effect of surface area to optimize the sensing properties towards CO. Three nanofibers were fabricated at calcination temperatures of 400, 500 and 600 °C. Increase in the calcination temperature caused a decrease in BET (Brunauer-Emmett-Teller) surface area and an increase in crystallite size. Measurements identified the optimum temperature of 300 °C where the highest response was obtained for INF (calcination = 400 °C) to 100 ppm CO. INF (calcination = 400 °C) showed faster response and higher sensitivity than other calcinations and commercial nanopowder. This happened due to the larger surface area created by lower calcination temperature that helped to adsorb more oxygen.

#### Gold Doped Indium Oxide

In a recent study, Fu et al. [92], the authors doped In_2_O_3_ with Au and observed its sensitivity for detecting CO at room temperature (25 °C). They created four Au/In_2_O_3_ hybrid nanomaterials corresponding to four different annealing temperatures (300, 400, 500 and 600 °C). It was reported that the lowest annealing temperature resulted in the highest response at room temperature and decreased with the increase in the operating temperature. The response increased according to the increase of CO concentration and decrease of humidity. The sensor response to CO was found to be 7–8 times higher than other tested gases that proved the selectivity of this sensor.

### 5.5. Tungsten Oxide (WO_3_)

**Figure 19:**
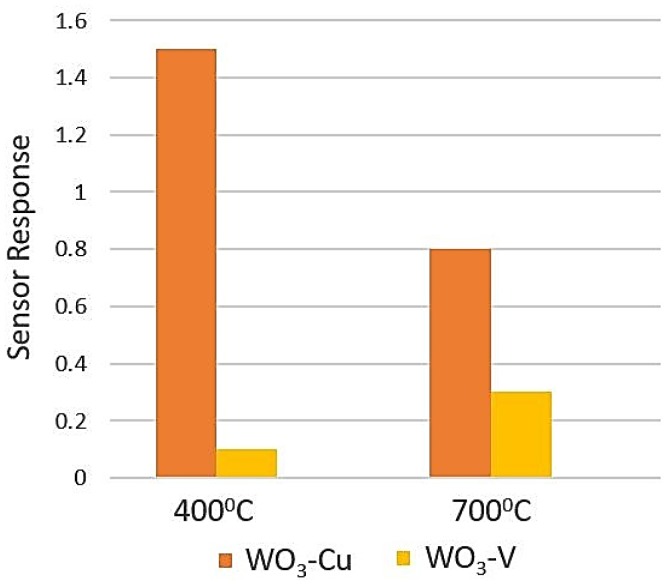
Sensor response of WO_3_-Cu (operating temperature 200 °C) and WO_3_-V (operating temperature 300 °C) annealed at 400 °C and 700 °C towards 100 ppm CO.

Some researchers found tungsten oxide (WO_3_) as a CO sensitive material. In [93], Hübner et al. tried to observe the sensitivity of WO_3_ towards CO gas as a function of oxygen at different gas concentration level. They prepared their sensing layer using sol-gel method. They used three different oxygen concentrations (one is below detection limit and other two are 150 and 22,000 ppm). They showed the sensor resistance dependency on time at different oxygen condition, by which it was evident that tungsten oxide can be used as CO gas sensitive material. However, they did not give any significant analysis on that. Jimenez et al. [94] conducted some research on the sensitivity of metal doped WO_3_ towards CO gas. They prepared 2% copper and vanadium catalyzed WO_3_ and annealed them at two different temperatures (400 °C and 700 °C). They found slightly better sensing response towards 100 ppm CO than 1 ppm NO_2_ gas. However, sensitivity towards NH_3_ gas was extremely better than both CO and NO_2_ gases. Figure 19 shows the sensor response of WO_3_-Cu at 200 °C and of WO_3_-V at 300 °C towards 100 ppm CO in synthetic air (80% N_2_ and 20% O_2_) condition.

## 6. P-Type Metal Oxides for CO Sensing

In p-type metal oxide semiconductors, the resistive core and semiconducting shell works parallelly, which is fully opposite as described in Section 5. For this reason, the conductivity of sensing film decreases, because of the increase of hole concentration in shell. Thus, the resistance of p-type MOS film increases. Recently investigated p-type metal oxide semiconductors for CO gas sensing application are Cobalt Oxide (CoO and Co_3_O_4_), Nickel Oxide (NiO) and Copper Oxide (CuO). Generally, p-type metal oxide semiconductors exhibit lower response to CO than n-type semiconductors [50,51].

### 6.1. Cobalt Oxide (CoO and Co_3_O_4_)

**Figure 20:**
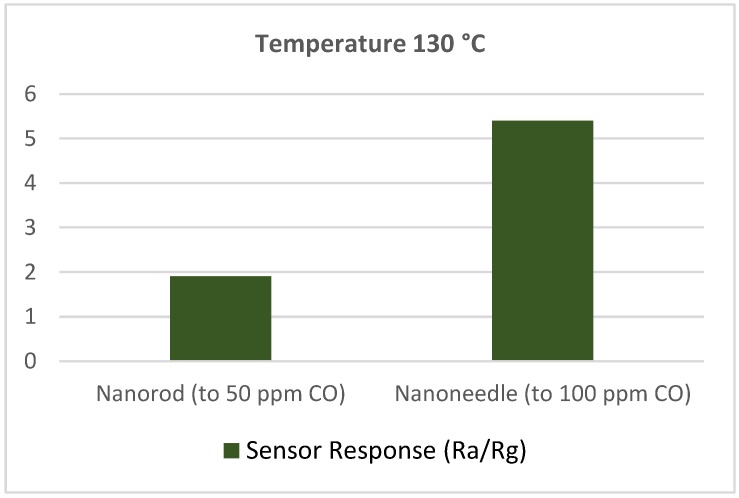
Response comparison between Co_3_O_4_ nanorod and nanoneedle.

Both cobalt oxides, CoO and Co_3_O_4_ can be used for CO sensing. However, Co_3_O_4_ is used. For example, Patil et al. [95] fabricated Co_3_O_4_ nanorods via the co-precipitation method. The radii of nanorods were varied in the range 3–4 nm. It was reported that the response of Co_3_O_4_ nanorods was higher than that of commercial Co_3_O_4_ powders for 5–50 ppm concentration of CO. They determined the optimum operating temperature to be 250 °C. The response and recovery times were also very fast, 3–4 s and 5–6 s, respectively. The response was much better for CO compared with other gases, including hydrogen, ethanol, carbon dioxide and liquid petroleum gas. They said that the higher surface-to volume ratio of Co_3_O_4_ nanorods and stronger bonding among nanoparticles helped to that high response towards very low amount of CO gas concentration. Wen et al. [96] tested Co_3_O_4_ nanoneedles for CO concentration of 100 ppm at 130 °C and observed the response shown in Figure 20. In another study [97], Co_3_O_4_ microrods were fabricated for CO gas sensing. They reported a sensitivity (~2) at 100 ppm CO concentration at 220 °C. Vetter et al. [98] investigated the operating temperature dependency of Co_3_O_4_ nanostructured sensors for low ppm CO gas concentrations. They measured the sensitivity, response and recovery times, and conductance change for two different operating temperatures (200 °C and 290 °C) at 50% RH. The sensor response at 1–25 ppm CO concentrations was better at temperature 200 °C, because of its very sharp conductance decrease.

#### 6.1.1. Gold-Palladium-Platinum Doped Cobalt Oxide

Nagai et al. [99] proposed the use of 3 wt.% AuPdPt doped CoO (APP-CoO) and Co_3_O_4_ (APP-Co_3_O_4_) as catalyst in thermoelectric CO gas sensing. They measured the voltage change for both catalysts for a range of 0–1000 ppm at 200 °C. They reported that APP-CoO showed better selectivity. 

#### 6.1.2. Tin Oxide Doped Cobalt Oxide

As n-type metal oxide semiconductors have better sensing properties towards CO than p-type semiconductors, then n-type semiconductor materials can be doped with p-type semiconductor materials for increasing its sensitivity in optimum conditions. In a recent study, Kim et al. [100] proposed n-type-p-type hetero-structured gas sensors. To improve the sensitivity of the p-type Co_3_O_4_, they prepared n-type SnO_2_ doping onto p-type and tested for CO gas sensing application, which gave them some great result towards very low CO gas concentration. They prepared SnO_2_-Co_3_O_4_ nanofibers that were calcinated at 600 °C. CO was exposed to five different SnO_2_-Co_3_O_4_ nanofibers having different SnO_2_/Co_3_O_4_ ratio for finding the optimum fabrication condition. They measured the response for 0.1SnO_2_-0.9Co_3_O_4_, 0.3SnO_2_-0.7Co_3_O_4_, 0.5SnO_2_-0.5Co_3_O_4_, 0.7SnO_2_-0.3Co_3_O_4_ and 0.9SnO_2_-0.1Co_3_O_4_ at an optimum temperature of 350 °C. The 0.5-0.5 composition had the highest response towards 1, 5, and 10 ppm CO. However, the 0.5SnO_2_-0.5Co_3_O_4_ showed very poor selectivity along with same amount of Sulphur dioxide, acetone and benzene at same temperature.

### 6.2. Nickel Oxide (NiO)

Khaleed et al. [101] fabricated nickel oxide (NiO) and nickel oxide with activated carbon (NiO-AC). The surface area of NiO-AC was greater than that of pure NiO. The authors measured the sensitivity as S = R_g_/R_ng_ × 100 where R_g_ and R_ng_ were resistance in gas and no-gas environments. They found that sensitivity and response times both were better for NiO-AC. The selectivity for CO gas was higher for NiO-AC compared to that for CH_4_ and NH_3_. This was because the large surface area enhanced the electrical conductivity of NiO-AC, which maximized the adsorption and desorption rates. For this reason, CO sensitivity was improved, and response time also became smaller. Both NiO and NiO-AC had very good selectivity compared to that for methane and ammonia.

### 6.3. Copper Oxide (CuO)

**Figure 21:**
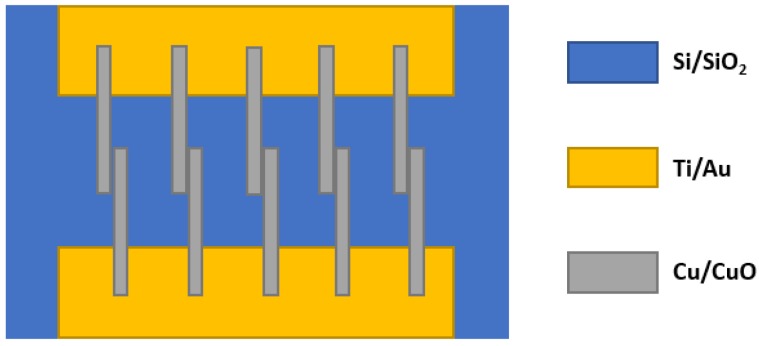
Top view of the fabricated CuO gas sensor.

**Figure 22:**
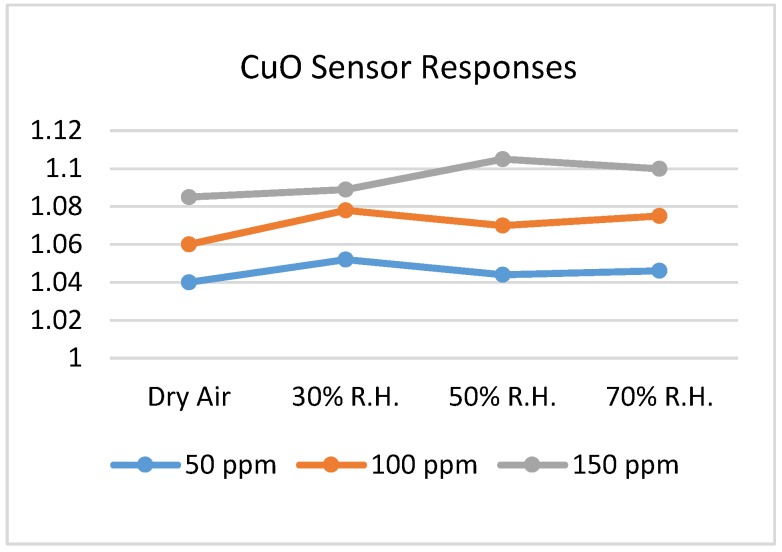
CuO sensor response for different humid levels and concentration levels at 325 °C.

Steinhauer et al. [102] synthesized copper oxide (CuO) nanowires by doing thermal oxidation of Cu microstructures. They investigated these nanowires towards CO concentration level 25–150 ppm under dry and different humid conditions (30%, 50% and 70% R.H.) to test the humidity effect upon the sensitivity. The authors prepared Ti/Au metallization layer on Si/SiO_2_ substrate and produced Cu layer for fabricating adjacent Cu lines. All preparations were done by lift-off process of thermally evaporated layers (Figure 21 shows the top view of the fabricated sample). Cu micro-lines were then thermally oxidized for making CuO nanowires and the whole sample was annealed for 3 h at 350 °C. They used synthetic air (80% N_2_ and 20% O_2_) for creating their testing condition. From the investigation, they found that the sensor resistances at 50% R.H. are higher than at dry synthetic air towards every CO concentration. Figure 22 shows another finding, which says that different humid levels do not create any significant variation on CuO sensor response (S = R_CO_/R_air_). Sensor responses at different humid levels are within 1.03–1.11 at 325 °C.

#### Copper Oxide (CuO) with Cerium Oxide (CeO_2_)

**Figure 23:**
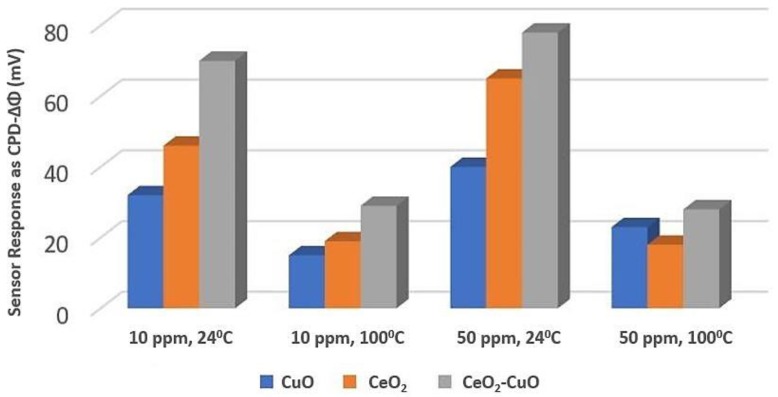
CeO_2_-CuO sensor response for different CO concentrations at room temperature and 100 °C.

Generally, both cerium oxide (CeO_2_) and copper oxide (CuO) are sensitive towards CO at higher operating temperatures, which is difficult to build up. For the improvement in CO sensing performance at room temperature, Tanvir et al. [103] made a mixture of equal amount of CeO_2_ and CuO nanoparticles (1:1) and determined the work function (Φ) readout towards CO by Kelvin probe measurement system. They prepared CeO_2_-CuO layer on top of TiN layer (backing electrode), which was on top of silicon substrate. They did surface CPD (contact potential difference) alternation with a reference electrode (here gold) for measuring the change in work function (ΔΦ). They observed that the sensor responses (CPD-ΔΦ) is better for the 1:1 CeO_2_-CuO mixture than pure CeO_2_ and CuO at room temperature (24 °C). They also found that all sensor responses were independent of gas concentration. Figure 23 shows the sensor performance enhancement towards CO at room temperature and 100 °C. They told that the decrease of amount of adsorbed water within the nanoparticles layer at high temperature can be a cause for lower sensor response than at room temperature.

## 7. Summary of MOS Based (except SnO_2_) CO Sensors

In Table 2, a summary on SnO_2_ based CO sensors was given. In this section, we present a summary of the other effective metal oxide semiconductor-based CO sensing techniques discussed in this review paper. For almost most of these techniques, we briefly list some of their advantages and limitations in Table 3 for an easy overview.

## 8. Optical Based CO Detection System

Aside from metal oxide based sensors, there have been proposed also optical based methods for detecting CO. Optical methods exploit the molecular absorption phenomena of CO gas within a specific range of light spectrum. For example, quantum cascade laser based and non-dispersive infrared (NDIR) based sensors were proposed in Reference [104]. The major disadvantages of the optical based methods include: (1) Bulky size and (2) high cost. That is why the optical based detection methods need more research before becoming commercialized at a large scale.

### 8.1. Quantum Cascade Laser Based Sensing

Kosterev et al. [105] introduced quantum cascade laser based quartz-enhanced photoacoustic spectroscopy for trace gas detection. They used Alpes lasers that operated at wavelength 4.55 µm. This approach works because the absorption is higher in mid-infrared zone than in near-infrared zone for low concentrations of CO. The method used a quartz tuning fork inside the gas chamber. Incident laser rays from the quantum cascade laser (QCL) were deflected and captured by an IR (infrared) detector. QCL was driven through function generator and QCL current driver. Photoacoustic (PA) wave was created through optical absorption and vibration-translation energy transfer within carbon monoxide molecular species. This photoacoustic signal vibrated the quartz tuning fork and this vibration was taken out through amplifier-lock-in circuit and processed data in PC. This data gave the detection result of CO gas. Here nitrogen gas was mixed with CO. They found very slow production of PA signal from this mixture at 32.8 kHz frequency, which proved its slow response time.

**Figure 24:**
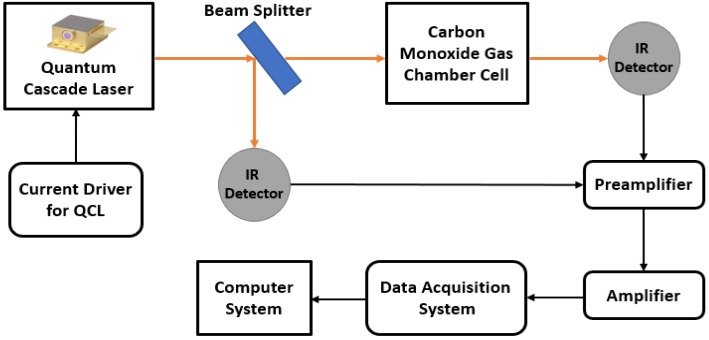
Quantum cascade laser based CO detection system using absorption methodology. QCL, quantum cascade laser.

In another study, Li et al. [106] fabricated a CO sensor with QCL for low CO concentrations. This method detects the ray after absorption via a photoconductive HgCdTe IR detector, which produce electrical signals as output. These signals were supplied to the PC via a pre-amplifier, amplifier, and data acquisition system. The set-up is shown in Figure 24. Signal processing techniques were used and response times under less than 10 s were achieved for 2 ppm concentration. As another example, Li et al. [107] proposed dual-spectroscopy technique that used QCL for detecting CO. They investigated two techniques: (i) Direct absorption spectroscopy (DAS) and (ii) Wavelength/frequency modulation spectroscopy (WMS). They reported that the sensing precision at 1 s was 6 ppb and 1.64 ppb for DAS and WMS, respectively.

### 8.2. Non-Dispersive Infrared (NDIR) Based Sensing

**Figure 25:**
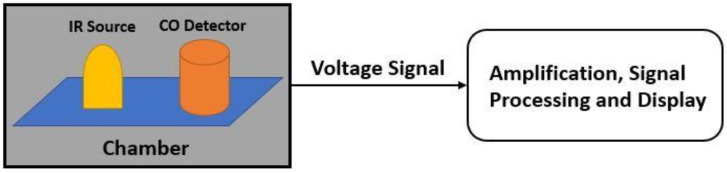
Non-dispersive infrared (NDIR) based CO detection system.

Tan et al. [108] introduced an infrared (IR) optical sensor-based detection system where non-dispersive infrared (NDIR) methodology was followed for a large range detection of CO at concentrations of 0–44,500 ppm. Their solution used a mid-infrared (MIR) light source whose light was reflected by the metal surface. The reflected light was detected by an integrated IR detector inside the gas chamber. Then, the detected signals were amplified and then processed by a micro-controller to calculate the response. The total NDIR based optical detection system is illustrated in Figure 25.

## 9. Conclusions and Summary

The main goal of this work is to review carbon monoxide gas sensors for using them in cyber physical systems, so that humans can quickly be alerted to avoid health related problems. It is necessary to determine which material will be more efficient, and which technique will be more beneficial for implementing this CPS framework. The main aim is to determine a CO sensor, which can show detection ability at a very low-level CO concentration under cost effective and achievable conditions. This paper gives the importance of CO gas sensing and necessity to implement CPS in CO gas detection system. However, for a better virtual-real world interaction-based system, we need a very well sensing device that can set the input of CPS. Through these inputs, the whole system can run and can interact with the physical world. That is why different kinds of CO sensors are extensively reviewed, compared and examined based on some key performance parameters, which can give a greater overview to select the proper CO sensing technology.

As discussed in this review article, several potential metal oxide semiconductors as gas sensors were investigated over the past recent years for their better sensing properties and selectivity towards carbon monoxide gas. Their performance was changed by many factors, such as film thickness, doping concentration, operating temperature, calcination temperature, gas concentration etc. Optimized doping of metals (Au, Cu, Pd, Pt, Ag, Al, In, Ga etc.) and some other selective materials (MWCNT, Graphene etc.) with metal oxide semiconductors can drastically affect the sensitivity, response time and selectivity. N-type metal oxide semiconductors were found much more effective as CO gas sensing material, though some p-type MOS also worked. Better understanding of these n-type materials properties and fabrication techniques, and their reasonably low cost make them more effective in this toxic gas monitoring. As a pure undoped material, tin oxide (SnO_2_) and titanium oxide (TiO_2_) have better sensitivity than all other pure metal oxide semiconductors to different amount of CO concentration. The highest sensitivity (84%) was found in microelectromechanical systems (MEMS) based gas sensors using titanium oxide. However, it needed an integrated microheater for attaining high operating temperature. Again, Pd and Pt doped tin oxide gave high response in room temperature, which can overcome this above situation. Similarly, every factor can be calibrated such a way that the sensor can be good and fast responsive and selective. For metal oxide techniques, a short and collective overview on their performances, advantages and limitations has been given in Table 2 and Table 3 so that it will be easier to decide what research should be done on them.

Besides these metal oxides, some optical based CO sensors were also discussed. These laser and infrared source based sensors were newly introduced, and they can easily surpass some problems of metal oxide semiconductors like high working temperature, doping etc. However, they have some of their own problems like bulkiness and high production cost. These optical based sensors need to be researched more to overcome these problems and can become a perfect candidate for commercialized CO detection system. Therefore, it can be concluded that a CPS-CO system will save lives instantly. Optimizing all the complexities, micromachined MEMS-based CO sensors using n-type metal oxides and optical-based CO sensors are the new frontiers for cyber-physical application based detection system.

## Figures and Tables

**Table 1:** Health problems according to carbon monoxide (CO) concentration and exposure time.

Concentration of CO (Exposure Time)	Created Health Problems
35 ppm (6–8 h), 100–200 ppm (2–3 h), 400 ppm (1–2 h), 800 ppm (45 min), 1600 ppm (20 min), 3200 ppm (5–10 min), 6400 ppm (1–2 min)	Headache, dizziness, nausea, loss of judgment and convulsions
1600 ppm (2 h), 3200 ppm (30 min), 6400 ppm (<20 min), 12,800 ppm (<3 min)	Respiratory arrest, severe conditions (coma) and death

**Table 2:** Brief summary of the SnO_2_ based MOS materials for CO detection.

Study	Material	Performance	Optimum Temperature	Advantages	Limitations
Kock et al. [55]	SnO_2_ nano wire	Response time 25 s	250 °C	Low concentration detection	Resistance suddenly increased
Lee et al. [56]	SnO_2_ thin film	Sensitivity 59%	270 °C	MEMS based structure and good stability	High CO concentration
Sharma et al. [57]	Cu-SnO_2_ thin film	Response time 5–10 s	320 °C	Fast response	High CO concentration and temperature
Menini et al. [58]	Pd-SnO_2_ thin film	Sensitivity ~80%, Response time ~50 min	450 °C	Very high sensitivity	Very slow detection and high operating temperature
Wang et al. [60]	Pt-SnO_2_ porous nano solid	Sensitivity (R_a_/R_g_) 64.5	Room temperature	Room temperature measurement and good selectivity	Humidity dependency
Manjula et al. [64]	Au-SnO_2_ thin film	Sensitivity ~90%	Room temperature	Room temperature measurement, good selectivity and no humidity effect	More doping caused decrease in response
Zhao et al. [65]	MWCNT-SnO_2_ nano particle	Sensitivity ~46%	300 °C	Fast detection and good stability	High operating temperature
Ghosh et al. [68]	Ca-SnO_2_ thin film	Difference of Response and Recovery ~25 s	350 °C	low CO concentration measurement, good selectivity and stability	High operating temperature
Nikan et al. [69]	ZnO-SnO_2_ thin film	Difference of response and recovery time ~11 min	300 °C	Very small doping concentration	Slow recovery and high operating temperature
Bai et al. [71]	CuO-SnO_2_ nano composite	Sensitivity (R_a_/R_g_) ~95	235 °C	Low concentration detection and good selective	High annealing temperature

**Table 3:** Summary of the MOS materials for CO detection.

Study	Material	Performance	Optimum Temperature	Advantages	Limitations
Rao et al. [80]	TiO_2_ thin film	Sensitivity 84%	500 °C	MEMS based structure, low concentration	Very high operating temperature
Lee et al. [81]	MWCNT-TiO_2_ thin film	Response time 4 s	300 °C	Fast and low concentration detection	High operating temperature
Hijri et al. [84]	Al-ZnO nano particle	Sensitivity (R_a_/R_g_) ~6	300 °C	Fast and low concentration detection	Optimum doping concentration varied
Gong et al. [85]	Cu-ZnO thin film	Difference of response and recovery time ~100 s	350 °C	Low concentration detection	High operating temperature
Wei et al. [86]	Pd-ZnO nano fiber	Response time 25–29 s, Recovery time 12–17 s	220 °C	Low concentration detection and good selectivity	Moderate sensitivity
Kuhaili et al. [89]	CeO_2_-ZnO thin film	Response time 44 s, Recovery time 40 s	380 °C	High resistance change	High CO concentration and high operating temperature
Choi et al. [90]	In_3_O_4_ micro spheres	Response time 4–8 s, Recovery time 5–10 s	400 °C	Fast and low concentration detection	High operating temperature
Fu et al. [92]	Au-In_2_O_3_ nano materials	Response ~9, response and recovery time ~30/30 s	25 °C	Room temperature measurement and very good selectivity	Influence of humidity and detection ability up to 100 ppm
Patil et al. [95]	Co_3_O_4_ nano rod	Response and Recovery time 3–4 and 5–6 s	250 °C	Fast, low ppm detection and good selective	Low resistance change
Steinhauer et al. [102]	CuO nano wires	Sensor Response (R_CO_/R_air_) ~1	325 °C	No dependency on humidity variation	High operating temperature
Tanvir et al. [103]	CeO_2_-CuO thin film	Sensor Response ~80 mV	24 °C	Room temperature measurement	It needs more investigations as a new material
